# Auditory Beat Stimulation and its Effects on Cognition and Mood States

**DOI:** 10.3389/fpsyt.2015.00070

**Published:** 2015-05-12

**Authors:** Leila Chaieb, Elke Caroline Wilpert, Thomas P. Reber, Juergen Fell

**Affiliations:** ^1^Department of Epileptology, University of Bonn, Bonn, Germany

**Keywords:** auditory beat stimulation, monaural beats, binaural beats, cognition, mood states, vigilance, memory, auditory steady-state response

## Abstract

Auditory beat stimulation may be a promising new tool for the manipulation of cognitive processes and the modulation of mood states. Here, we aim to review the literature examining the most current applications of auditory beat stimulation and its targets. We give a brief overview of research on auditory steady-state responses and its relationship to auditory beat stimulation (ABS). We have summarized relevant studies investigating the neurophysiological changes related to ABS and how they impact upon the design of appropriate stimulation protocols. Focusing on binaural-beat stimulation, we then discuss the role of monaural- and binaural-beat frequencies in cognition and mood states, in addition to their efficacy in targeting disease symptoms. We aim to highlight important points concerning stimulation parameters and try to address why there are often contradictory findings with regard to the outcomes of ABS.

## Introduction

Auditory beat stimulation (ABS) has long been of interest for a wide array of applications, ranging from investigating the auditory steady-state response (ASSR) and measuring audiometric parameters in the brain, to understanding mechanisms of sound localization (1). In addition to this, a few studies also suggest that ABS can be used to modulate cognition (2), to reduce anxiety levels, as well as to enhance mood states (3). Other clinical targets also include traumatic brain injury (4) and attention-deficit hyperactivity disorder (5). Resulting studies have reported contradictory findings as to the effects of applied monaural-beat frequencies and binaural-beat frequencies, which have somewhat hampered the progress of further investigations addressing potential effects on cognition and mood effects amongst other possible targets. Here, we review relevant studies and look to highlight the most promising directions for future approaches.

## Literature Search Strategy

This review was conducted using the Preferred Reporting Items for Systematic Reviews and Meta-Analyses (PRISMA) criteria (6). The electronic databases PubMed and MEDLINE were searched initially using the single search terms “auditory beat stimulation, monaural beat, binaural beat, auditory steady-state response,” and then the combination of these search terms with the terms “cognition, memory, attention, mood, vigilance, anxiety, and creativity.” The strategy was not limited to human studies. In addition to the electronic search strategy, the reference lists of the manuscripts that were reviewed were examined to identify any additional articles not captured by the main search strategy. A total of 920 articles were identified in the initial search. Analysis of the papers followed the inclusion and exclusion criteria recommended by the PRISMA Guidelines. Articles that presented a combination of at least two terms from the list of search terms, “auditory beat stimulation, monaural beat, binaural beat, cognition, memory, attention, mood, vigilance, anxiety and creativity” were included. Manuscripts in English, original articles, and experimental studies were considered. Exclusion criteria were other study designs (case reports and case series), non-original studies including editorials, book reviews, and letters to the editor, and studies not specifically designed and focused on monaural and/or binaural-beat stimulation. Abstracts were screened for relevance and then full texts were assessed against inclusion criteria. After screening, a total of 30 articles were selected. During the construction of the manuscript, several other references were added, mainly referring to basic neurophysiological findings.

## Auditory Beat Stimulation

**Figure 1:**
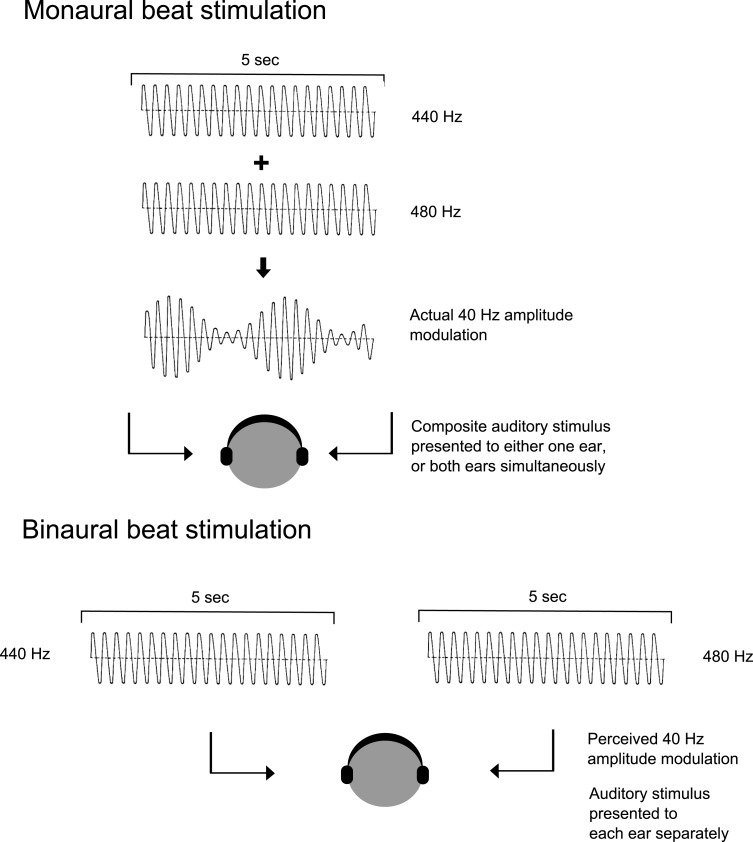
**Application of monaural and binaural beats**. The superposition of amplitude modulated signals of nearby frequencies delivered either to one ear or both ears simultaneously (monaural beats), or neighbouring frequencies to each ear separately (binaural beats) are shown here. Carrier tones of 440 and 480 Hz generating a 40 Hz beat is shown here as an example [Courtesy of Becher et al. (41)].

The effects of ABS have been predominantly investigated using monaural and binaural beats (Figure 1). The main differences between binaural and monaural beats are listed in Table 1. Monaural and binaural beats are generated when sine waves of neighboring frequencies and with stable amplitudes are presented to either both ears simultaneously (monaural beats) or to each ear separately (binaural beats). Monaural beats are physical beats, which are objectively heard when the combination of two sine waves at neighboring frequencies (e.g., 400 and 440 Hz) are summated and presented to each ear at the same time resulting in an amplitude modulated (AM) signal. The beat corresponds to the difference between the two frequencies (in this case 40 Hz). Binaural beats are generated when the sine waves within a close range are presented to each ear separately. For example, when the 400 Hz tone is presented to the left ear and the 440 Hz tone to the right, a beat of 40 Hz is perceived, which appears subjectively to be located “inside” the head. This is the binaural beat percept. The binaural beat percept was first reported by H. W. Dove in 1839 and outlined in detail by Oster over five decades ago (7). Oster reported that the binaural beats were detected only when the carrier frequency was below 1000 Hz, a finding that confirmed an earlier study by Licklider and colleagues (8). This indicates that beat carrier frequencies have to be sufficiently low enough to be temporally encoded by the cortex (9).

**Table 1:** **Monaural and binaural-beat stimulation: main characteristics**.

Monaural beats	Binaural beats
Physical/objective beat	Subjective percept
Presentation of composite frequencies to one ear or both ears simultaneously	Presentation of neighboring frequencies to each ear separately
Peripheral	Central
Demodulated in the cochlea	Processed in the medial superior olivary nuclei
Able to be perceived in either one or both ears	Require combined action of both ears
Heard across a wider beat frequency range and at higher carrier tones	Present when beat frequencies are low and with carrier tones below 1000 Hz

*The main differences between monaural and binaural beats are summarized and outlined*.

## Neurophysiology of Auditory Beat Processing

Acoustic stimuli are heard when the peripheral components of the auditory pathway (ears, cochlea, and inner hair cells) convert pressure waves into neural action potentials via mechano-electrical transduction. This is the first order of auditory processing prior to sound waves being encoded (or rather re-encoded) by the primary auditory cortex. Auditory information is further processed at a number of subcortical structures. Auditory nerve fibers leaving the cochlea converge with the vestibulocochlear nerve and enter the cochlear nucleus (CN) in the brainstem and bifurcate. As the nerve fibers branch they form synapses with different subtypes of neurons – spherical bushy cells, globular bushy cells, and stellate cells, each of which differ in their temporal and spectral response properties (10). Information is then relayed to either the inferior colliculus (IC) via outputs from the stellate and dorsal cochlear nucleus (DCN) cells, or by an indirect route to the superior olivary complex (SOC). Bushy cells of the anteroventral cochlear nucleus (AVCN) project outputs via this route (11). The SOC processes convergent information from the left and right ears and cues related to sound localization (12). The left and right IC has a commissural connection, which allows for binaural interactions within the ascending pathway, and is comprised of numerous subnuclei, the largest of which is the central nucleus (ICC) (13, 14). Here, the temporal integration window between the IC and the auditory cortex enables processing of monaural characteristics such as amplitude modulation (15). From here, outputs travel to the medial geniculate nucleus (MGN) of the thalamus, where thalamic output fibers connect to the auditory cortex located in the temporal lobes (13, 14).

The neurophysiological processing of binaural and monaural beats differs slightly. Draganova and colleagues underlined these differences by referring to monaural beats as “peripheral” – as they interacted at the cochlear level, and binaural beats as “central,” i.e., the binaural beat percept being the result of the effect of a central interaction which mostly likely occurs in the superior olivary nuclei (16). Monaural beats are heard when a composite auditory stimulus is presented to both ears simultaneously, which is detected by the cochlear and relayed to the brain stem and auditory cortex. Binaural beats, however, are only subjectively perceived when two sine waves of nearby frequencies are delivered to each ear separately. Brainstem neurons in the SOC, which are sensitive to phase shifts between both ears, fire action potentials at a rate corresponding to the phase difference between both ears and generate the binaural-beat percept. Thus the binaural-beat percept is caused by the major neural mechanism which enables sound localization (1).

## The Auditory Steady-State Response

The ASSR is a composite auditory evoked potential which can be elicited using repetitive acoustic stimuli which continually persist over a time period. The ASSR follows the envelope of a complex stimulus, and it has been suggested that the steady-state response drives the background activity of the EEG (17). Regan defines the steady-state response as “an evoked potential whose constituent discrete frequency components remain constant in amplitude and phase over an extended time period” (18). In a seminal study, Galambos and colleagues recorded click-related potentials (ERPs) with latencies between 8 and 80 ms post stimulus onset extracted from the EEG recorded from electrodes placed at the forehead and ear. These ERPs are termed the middle latency (MLR) responses, and can be acquired after the earlier brainstem responses. It was observed that the ERP was most evident when the clicks were delivered at a rate of 40/s, a response that they subsequently named the 40 Hz ERP [which is a composite of the several transient waves comprising the MLR (19)]. As the steady-state response consists of the superposition of the subcomponents of the MLR, studies have sought to predict the steady-state response from its transients (20). However, the prediction of the steady-state response from the superposition of its transient waveforms is not always accurate, and therefore provides evidence of oscillatory entrainment (17). Since this early study, many more have investigated ASSRs with the aim of examining their role in attentional processes (21–23) and possible clinical applications (24, 25). In a study examining the effect of selective attention on the 40 Hz transient response, Tiitinen et al. (21) demonstrated that the 40 Hz transient response was larger over frontal and central areas when participants were told to pay attention to tone stimuli, than when they were instructed to ignore them. This demonstrated that the transient 40 Hz response is enhanced under active attention. In a later study, Ross and colleagues (22) showed that the ASSR was also enhanced under conditions of active attention. Participants performed a modulatory discrimination task in which they had to indicate when the standard stimuli presented (40 Hz AM tones) changed in modulation frequency (to 30 Hz, the target stimuli). Auditory stimuli were delivered monaurally to the right ear. Magnetoencephalography (MEG) recordings showed that the ASSR was enhanced during auditory attention, and was more pronounced in the left hemisphere contralateral to stimulation (22). Another study reported different ASSR patterns to attended versus unattended AM tones (20 and 45 Hz) delivered to the left and right ear simultaneously (26). The authors reported an attention-dependent modulation of the ASSR patterns only for the 20 Hz stimulation, but not for the 45 Hz stimulation. Together, these findings indicate that directed attention has an impact on ASSR amplitudes, but that these effects likely dependent on modulation frequency.

## Auditory Steady-State Responses to Monaural and Binaural Beats

Beat stimulation has been used to examine the source and origin of the ASSR, an often controversial debate due to contradictory findings (27, 28). More recent studies have sought to clarify this issue. For example, the 40 Hz ERP has been used to investigate the cortical sources of the ASSR, as well as the characteristics and effects of monaural and binaural-beat stimulation frequencies. Ross and colleagues recorded ASSRs to AM tones with modulation frequencies of ~40 Hz using MEG. They reported that ASSR amplitudes decreased with increasing carrier frequencies, with the ASSR amplitude at 250 Hz to be a magnitude of three times larger than at 4000 Hz. Importantly, they observed that when beats at 39 and 41 Hz are simultaneously presented both beats can be perceived at the same time (29).

To probe the cortical representation of binaural-beat frequencies, Karino et al. applied modulation frequencies of 4.00–6.66 Hz while recording magnetic fields using MEG. The authors reported that the binaural beat ASSR arose from the superior temporal, posterior parietal, and frontal cortices, in addition to the auditory cortex (30). Another study applied a similar technique to that of Pantev et al. (31), by comparing a transient of the MLR–N1m to ASSR responses to monaural and binaural-beat stimuli, recorded using MEG (16). Their findings showed that ASSR to both monaural- and binaural-beat stimuli are located anterior and medially to Heschl’s gyri within the Sylvian fissure, and when compared with the N1m source, place the ASSR generating network within the primary auditory cortex, which is also in line with other studies (29, 32). The authors also observed that the magnetic field amplitudes of the ASSR elicited by monaural beats were ~5 greater than those of the ASSR to binaural beats (16). A recent study has also reported similar findings concerning the magnitude of responses to monaural and binaural beats, and that stimulation conditions were reflected in interhemispheric phase differences (33). Schwartz and Taylor also reported a lesser ASSR amplitude response to binaural-beat stimuli compared to monaural beats. A 40 Hz binaural beat ASSR was evoked with a carrier frequency at 400 Hz but was undetectable above 3000 Hz. This was not the case for the monaural beat stimulation frequencies, which could be detected above 3000 Hz (9).

Two studies by Pratt and colleagues have examined cortical evoked potentials first to binaural-beat frequencies, and then a following study reported responses to monaural beats. The first study aimed to explore ERP responses to binaural beats of 3 and 6 Hz using two different carrier frequencies of 250 and 1000 Hz. Similar to other studies (29), the authors reported that beat-evoked responses were higher in amplitude to beats with a low carrier frequency (250 Hz) and also to the lower beat frequency itself (3 Hz). In the second study, Pratt et al. (34) recorded beat-evoked ERPs again, this time to both monaural and binaural beats at the same frequencies (3 and 6 Hz), as well as at the same base carrier frequencies (250 and 1000 Hz), but using different onset phases, i.e., by applying monaural beats that differed in phase by a quarter of the beat period. The amplitudes of the beat-evoked oscillations were higher in response to monaural beats and also to the lower carrier and beat frequencies (250 and 3 Hz). The sources of the beat-evoked responses both to the monaural and binaural-beat stimuli originated in the temporal lobe regions and were lateralized to the left hemisphere, regardless of the difference in phase-onset between the stimuli. These findings suggest that the processing of binaural and monaural beats occurs in the same cortical regions regardless of the onset phase (34).

In a study aiming to map the origin of the ASSR, Pastor and colleagues applied a train of monaural stimuli at 12 different stimulation rates. Using positron emission tomography [(PET)-H_2_^15^O] and EEG data, the authors reported an increase in regional cerebral blood flow (rCBF) together with oscillatory responses peaking at 40 Hz. These findings indicate that the ASSR is related to an increase in overall synaptic activity in the auditory cortex at this frequency, and is not just due the superposition of MLRs (35). Interestingly, an earlier study by Pantev et al. compared the transients of the MLR and the 40 Hz SSR, and found that the two evoked responses originate from different sources in the auditory cortex, which were tonotopically segregated with regard to carrier frequencies (31).

Altogether, these findings suggest that binaural and monaural beats are processed in the same cortical areas, and that the beat-generated ASSR is related to changes in synaptic activity in the auditory cortex as well as to the superposition of MLR transients, also originating in the primary auditory cortex.

## Frequency-Specific EEG Changes Due to Beat Stimulation

Studies reporting significant effects after application of binaural beats using continuous EEG have shown changes in only certain frequency bands, these include gamma (9, 35) and alpha (36). A recent study seeking to investigate these effects in the alpha and beta ranges has reported no significant changes. Vernon and colleagues applied binaural beats in the alpha (10 Hz) and beta (20 Hz) frequency ranges for 1 min duration over ten trials to evaluate whether a frequency following response (FFR) could be elicited to these frequencies in two separate participant groups. Each trial was interleaved with exposure to a pure tone played at 400 Hz while EEG was recorded from the left and right temporal regions. The authors observed a slight decrease in resting baseline amplitudes for both beat frequencies, from the pre- to post-entrainment and during the experimental session, and participants exhibited reduced alpha activity during the binaural beat on-phase compared to the off-phase (36). In a similar study, Gao et al. (37) investigated the effects of binaural beats applied for a 5 min duration at 1, 5, 10, and 20 Hz. To detect EEG changes due to binaural beats, they analyzed relative power (RP), phase locking values (PLVs), and cross-mutual information (CMI). Relative power in the theta and alpha bands increased during delta and alpha binaural-beat frequency stimulation while it decreased in the beta band. During alpha and delta binaural-beat stimulation, reduction in CMI was detected among right temporal, frontal, and occipital areas 3.5 min after stimulation onset. However, during beta-beat stimulation, an increase and subsequent decrease in CMI was observed – this occurred between the left temporal and frontal areas (increase), and between right temporal and centro-parietal areas (decrease), and in the case of theta beat stimulation, an increase over left temporal and central cortical areas was observed. These data suggest that application of binaural beats in theta, alpha, delta, and beta frequencies is able to alter functional connectivity between brain regions (37).

Another study looking at the effect of putatively inhibitory and facilitatory binaural-beat frequencies (15 and 7 Hz, respectively) on meditation practice, reported significant entrainment effects (38). Application of binaural beats at a theta frequency (7 Hz) increased left temporal lobe delta power in experienced meditators, whereas this effect was not recorded in the novice participant group. When the beta beat frequency was applied novice participants showed more gamma power increase during meditation than the experienced mediator group (38). These results indicate that the effects of binaural beats depend upon prior experience and individual skills.

## ABS and Phase Synchronization

A study investigating binaural and monaural beat ASSRs compared the phases of the 40 Hz sinusoids derived from EEG recordings at various electrode locations (9). The authors observed a fronto-occipital phase shift in both the binaural and monaural beat ASSRs, of ~3–7 msec. These phase shifts indicate that the monaural and binaural beat ASSRs are generated by more than one neuronal network at different locations. The authors suggest that either the rostro-caudal phase shift of gamma oscillations may demonstrate a sequential recruitment of relevant cortical regions, similar to findings from a study looking at thalamocortical oscillations (39), or that the data could have been generated by more than one source with different orientations (9). Bilateral phase differences could be detected for the binaural beat, while monaural beats evoked responses of equal phase in both left and right hemispheres (33). The phase delays reported by Ross et al. and Schwarz and Taylor (9) may also reflect alterations in phase synchronization. Phase synchronization occurs when oscillations in two brain regions have a constant phase relation over some time period. Phase synchronization is integral to cognition as it supports the processes of neural communication, neuroplasticity and memory formation (40). If auditory beats do indeed induce phase synchronization, this may indicate a role for monaural and binaural beats in modulating memory processes. Recent findings based on intracranial EEG recordings in humans suggest that auditory beats are able to specifically alter not only EEG power, but also phase synchronization. For instance, an increased temporo-lateral phase synchronization was observed due to 5 Hz binaural-beat stimulation, while a decreased mediotemporal synchronization was detected during 5 Hz monaural beats (41).

## Studies Examining the Effects of ABS

The application of ABS to manipulate cognitive processes or for the modulation of mood or pain responses has been investigated, but has yielded contradictory results, especially with regard to binaural beats. Studies that reported statistically significant effects state that they are often weak and short-lived, and in addition, there is very little discussion as to which mechanisms are involved in the generation of these effects. This may be in part due to the nature of the stimuli itself, i.e., the binaural beat being a weak percept, and that most studies did not employ the use of measurement techniques like EEG or MEG to quantify resulting electrophysiological effects. Another possible reason for the reported inconsistencies may be due to the incomparability of the methodological approaches.

## Cognitive Effects of ABS

### Memory

Two studies applying binaural-beat stimulation at the theta frequency reported opposite effects on memory (2, 42). Wahbeh and colleagues tested verbal memory performance using the Rey Auditory Verbal Learning Test. Participants are asked to repeat a list of 15 unrelated words over several different trials. The test reflects both working and long-term memory processes (43). They reported that binaural-beat stimulation at 7 Hz, for a single 30-min session, resulted in decrease in immediate verbal memory recall in the experimental condition compared to control condition (42). In contrast, application of 5 Hz binaural-beat stimulation for 15 min, twice per day for 15 days, resulted in a significant increase in the number of words recalled post-stimulation, as measured using the Wechsler III Memory Scale, when compared to the other stimulation conditions (13 Hz binaural beats and a white noise control condition) (2). The Wechsler II Memory Scale assesses different working and long-term memory functions using a battery of tests. In this study, immediate recall of word lists was assessed (44). These results may suggest that prolonged exposure to ABS may affect verbal memory recall.

### Creativity

Creativity has been suggested to be related to divergent thinking, as opposed to convergent thinking. Divergent thinking refers to the generation of multiple answers to a given problem, while convergent thinking means aiming for a single, correct solution to a problem (45). A recent study reported positive effects of binaural beats applied at the alpha (10 Hz) and gamma (40 Hz) ranges on creativity. Creativity was assessed using the divergent thinking [Alternate Uses Task (AUT)] and convergent thinking [Remote Associations Task (RAT)] tasks, which were correlated with the spontaneous Eye Blink Rate (EBR), a marker of dopamine levels in the brain. The divergent thinking task (AUT) involved participants being asked to name as many uses for certain household objects as they possibly could. The task assesses four components: originality, fluency, flexibility, and elaboration. In the convergent thinking task (RAT), participants were required to name a single compound word which matched three seemingly unrelated words. In addition to these tasks, participants were also required to fill out a Positive and Negative Affect Scale questionnaire. Beat stimuli were applied for 3 min prior to the tasks. The results of this study indicated that binaural beats at both frequencies affected performance in the divergent, but not convergent thinking tasks. Authors noted that participants with a low EBR benefited from alpha binaural-beat stimulation, whereas those with high EBRs were either unaffected or impaired by alpha and gamma binaural-beat stimulation. In this study, background white noise was also added to each stimulus in order to amplify the binaural-beat percept (46).

### Attention

In a pilot study, Kennel et al. investigated the potential use of binaural-beat stimulation to reduce the symptom of inattention in children and adolescents with attention-deficit/hyperactivity disorder (ADHD) (5). ADHD is a developmental neuropsychiatric disorder diagnosed in children and adolescents. Individuals affected by ADHD exhibit the core symptoms of inattention, hyperactivity, and impulsivity in varying degrees of severity (47). Participants were either required to listen to commercial recordings of binaural beats embedded in natural sounds or a sham recording containing pink noise for 20 min, three times a week for 3 week duration. The Test of Variables of Attention (TOVA) and the Children’s Color Trails Test 1 and 2 (CCTT1 and 2) were performed to measure changes in attention over time and course of treatment. For this study the binaural-beat stimulation did not have a significant impact on attention, but participants reported subjectively experiencing less problems associated with inattention during the study period (5). Unfortunately, one of the main limitations of this study was that the beat stimulation parameters of the audio program administered were not reported.

## Anxiety, Mood States and Vigilance as Targets of ABS

### Anxiety

Two types of anxiety can be differentiated. State anxiety is a temporary increase in anxiety levels related to an event or situation. Trait anxiety, however, is a continually heightened level of anxiety which is a personal characteristic (48). In an interesting study, Padmanabhan and colleagues applied binaural-beat stimulation to patients suffering from pre-operative anxiety (3). Patients were assessed using the State-Trait Anxiety Inventory (STA-I) questionnaire, and beat stimuli were administered via a compact disk player with either binaural beats or a sham-like audio recording. The recordings contained binaural beat recordings within a delta frequency range. The authors reported a 26.3% decline in anxiety scores in the post-stimulation STA-I assessment for the binaural beat audio group when compared to a 11.1% decline in the placebo audio group (3). A similar approach was taken by Weiland et al. who used sound compositions of either natural settings, or with an embedded binaural-beat frequency of 10 Hz (49). The intervention was applied for 20 min and patients were requested to complete the STA-I in order to assess anxiety scores. They reported significant decreases in anxiety scores post-intervention in those patients who received the binaural-beat stimulation compared to the patient group who did not (49). Le Scouarnec et al. used a commercial binaural-beat recording for a pilot study examining levels of anxiety (50). Participants with mild anxiety disorders were asked to listen to a recording of binaural beat stimuli in the delta/theta range, daily for 30 min for a total of 1 month while detailing their anxiety ratings before and post-treatment using STAI-I scores. The authors reported that patients recorded a reduction in anxiety ratings and an increase in the number of times the patients chose to listen to the recordings (50). In a later study, it was also reported that patients who received binaural-beat stimulation in the delta frequency for 30 min daily over 60 days showed a significant decrease in trait anxiety scores assessed with the STA-I (51).

### Mood States

Mood is a temporary, conscious state of mind or predominant feeling (52). Mood states are often dependent upon external factors. Several studies have sought to modulate mood states by binaural-beat stimulation. Two studies by Wahbeh and colleagues looked at the effect of binaural beats at theta (7 Hz) and delta (0–4 Hz) frequencies on mood states (42, 51). Binaural beats were presented either daily over 60 days (at delta frequency) or once for 30 min (at theta frequency). Changes in mood states were assessed using the Profile of Mood States (POMS) questionnaire, given before and after stimulation. The POMS is a 65-item self-report questionnaire that contains total mood score and six subscales: Tension–Anxiety, Depression–Dejection, Fatigue–Inertia, Anger–Hostility, Vigor–Activity, and Confusion–Bewilderment (53). They reported a decrease in total mood disturbance, as well as a decrease in tension, anxiety, confusion, and fatigue subscales after delta beat stimulation compared the control condition. However, there was an increase in the depression and vigor subscales (51). In the second study, after 30 min of theta binaural-beat stimulation there was an increase of the POMS depression subscale in the experimental condition relative to the control condition (42). In a similar study, Lane et al. (54) reported decreases in POMS depression subscales after binaural-beat stimulation in the beta range (16 and 24 Hz), compared to presentation of beats in the theta/delta range (1.5 and 4 Hz). The authors suggested that perception of beta frequency beats is associated with less negative mood (54).

### Vigilance

Vigilance is the ability to maintain focus of attention and to remain alert to stimuli over prolonged periods of time (55). Vigilance tasks typically comprise of monotonic sensory processing requiring continuous attention. A recent study aimed to explore the impact of binaural beats on vigilance and personality traits assessed according to the Five Factor Model (FFM) (56). The FFM of personality identifies five traits: Neuroticism (N), Extraversion (E), Openness to experience (O), Agreeableness (A), and Contentiousness (C). A previous study had identified a correlation between O and C personality traits and cortical entrainment in the theta and beta bands using photic driving (57). Binaural-beat stimuli were applied at theta (7 Hz) and beta (16 Hz) frequencies while participants performed a vigilance task, in which they were required to respond to the presentation of a target stimulus (a number or letter), presented on a computer screen out of a list of serially presented stimuli, with a button press. EEG was recorded throughout the experiment and binaural beats were applied for a total of 4 min during the execution of the task. A baseline of white noise was played in between each stimulation epoch. The authors hypothesized that beats within the beta range would sustain or increase levels of vigilance (indicated by a reduction in response time during the vigilance task), based on earlier studies (54). In line with previous findings (57), they also suggested that O and C personality traits would be more susceptible to entrainment in the theta and beta beat frequency ranges, and that individuals who scored higher in trait category A would demonstrate higher beta power in the left temporal and central cortical regions (58). The authors reported no significant effects of the stimulation frequencies, either on vigilance or any interaction with personality. They suggested the short duration exposure to the binaural-beat stimuli was insufficient to alter vigilance or entrain cortical frequencies.

However, another investigation also examining the effects of binaural beats on vigilance and mood, reported significant effects. Lane et al. applied binaural beats at beta (16 and 24 Hz) and theta/delta (1.5 and 4 Hz) ranges for 30 min throughout a psychomotor vigilance task. The authors reported that beats in the beta range were associated with a less negative mood (see above) and improved performance in a vigilance task. However, the beat stimuli presented in this study contained a background of pink noise and had lower carrier tones to that of the previous study reporting negative effects of binaural-beat frequencies on vigilance (54). It may be that the choice of carrier tone impacts upon the efficacy of beat stimulation, as it was reported that lower carrier tones as well as beat frequencies produce more robust effects (34). The addition of a pink or white noise background may also have had an effect upon the processing of the beat percept (46). These differences, along with the length of the stimulation duration may account for the discrepancies seen in the outcomes of these similar studies.

## Conclusion

This review has aimed to give a brief overview of ABS and its role in cognition and potential use as a therapeutic tool for modulating mood states. While findings for most putative applications up to now are either solitary or contradictory, several studies consistently report a diminishing impact of binaural-beat stimulation on anxiety levels. The underlying neural mechanisms are still yet to be unraveled. Understanding how and where the binaural-beat percept is generated and which cortical networks are most affected will aid in the optimization of both monaural and binaural-beat stimulation as a tool to modulate cognitive and mode states. Many studies employing ABS as either a mechanistic tool or potential therapeutic aid, report contrasting findings. Further research, including more accurate reporting of experimental protocols, especially those studies undertaken in a clinical setting, will help to clarify the most promising effects. In a recent study, Ross and colleagues reported that inconsistencies relating to monaural and binaural beats at low frequencies, as well as at the 40 Hz frequency, could possibly be attributed to earlier investigations suggesting that they share common neural mechanisms (33). Many factors may impact upon the efficacy of beat stimulation, including the duration of the applied stimulus. Carrier frequencies may also play a role, as well as the addition of background white or pink noise, which may amplify the beat percept (46).

A study examining the effects of aging showed that regardless of age, a binaural-beat percept in the gamma range could be detected, but with less accuracy by older individuals (59). Some investigations also reported gender differences concerning binaural-beat perception (7) and alterations in auditory perception during the menstrual cycle (60). Other studies suggest that attending to the stimulus may play role (9). As many factors impact upon the efficacy of monaural and binaural-beat stimulation, a more in-depth reporting of beat stimulation parameters and protocols would offer the possibility to limit the methodological inconsistencies that may explain many of the contradictory outcomes reported in the literature. Most importantly, electrophysiological investigations comparing the effects of auditory beats under different stimulation conditions and parameters are still rare. Such studies are necessary as a fundament to allow the development of mechanistic hypotheses explaining the behavioral outcomes of beat stimulation.

## Conflict of Interest Statement

The authors declare that the research was conducted in the absence of any commercial or financial relationships that could be construed as a potential conflict of interest.
